# Is ball-possession style more physically demanding than counter-attacking? The influence of playing style on match performance in professional soccer

**DOI:** 10.3389/fpsyg.2023.1197039

**Published:** 2023-07-07

**Authors:** Leon Forcher, Leander Forcher, Hagen Wäsche, Darko Jekauc, Alexander Woll, Timo Gross, Stefan Altmann

**Affiliations:** ^1^Institute of Sports and Sports Science, Karlsruhe Institute of Technology, Karlsruhe, Germany; ^2^TSG Hoffenheim, Zuzenhausen, Germany; ^3^TSG ResearchLab gGmbH, Zuzenhausen, Germany

**Keywords:** football, team sports, tactics, athletic, running performance, trainer

## Abstract

In soccer, the offensive style of play describes characteristic behavioral features of the players at team level during the offensive phase of matches. This study aimed to investigate the effect of offensive playing style (i.e., while in ball possession) on physical and technical match performance during offensive play as well as success-related factors. The sample consisted of official tracking and event data of 153 matches of the 2020/21 German Bundesliga season. For every team in every match an offensive playing style coefficient was calculated to locate teams on a continuum between ball possession and counter-attacking style. This calculation contains 11 technical and physical performance parameters and has already been validated. In addition, dependent physical (e.g., sprinting distance), technical (e.g., passes), and success-related (e.g., goals) variables were examined. A separate linear mixed model was calculated for each dependent variable. While teams with lower playing style coefficient values (= counter-attacking style) covered more high-intensity (*p* ≤ 0.01; *R*^2^ = 0.13) and sprinting distances per second in possession (*p* ≤ 0.01; *R*^2^ = 0.14), teams with higher playing style coefficient values (= ball possession style) were physically more demanded over a whole match (e.g., more accelerations (*p* ≤ 0.01; *R*^2^ = 0.69), decelerations (*p* ≤ 0.01; *R*^2^ = 0.69), high-intensity (*p* ≤ 0.01; *R*^2^ = 0.36), sprint distance (*p* = 0.03; *R*^2^ = 0.08)). Furthermore, teams with higher playing style coefficient values played more horizontal passes (*p* ≤ 0.01; *R*^2^ = 0.73) and revealed better passing success rates (*p* ≤ 0.01; *R*^2^ = 0.17). In contrast, teams with lower playing style coefficient values played more long passes (*p* < 0.01; *R*^2^ = 0.58). The influence of the playing style coefficient on success-related variables was smaller (*p* ≤ 0.36; *R*^2^ = 0.10–0.13). Concluding, offensive playing style affects physical and technical match performance, but has limited influence on success. Hence, coaches can use the findings to optimize training contents to prepare players for the physical demands of a match.

## 1. Introduction

Match performance in soccer primarily consists of physical, technical, and tactical components (Sarmento et al., 2014). With the increasing availability of big data in professional soccer, this match performance can now be well quantified within its components (Forcher et al., 2022a,e). For instance, from a physical perspective, professional players run between 10 and 13 km per match while only sprinting 2–3% of this distance (Stølen et al., 2005; Sarmento et al., 2014). Furthermore, from a technical point of view, players are in ball possession 57 times and play 38 passes on average (Forcher et al., 2022d). Lastly, in a tactical context, studies revealed that passes with a higher potential of disrupting the opposing team lead to more successful attacks (Kempe and Goes, 2019; Forcher et al., 2021).

This physical, technical, and tactical match performance is influenced by a variety of contextual factors (Lago and Martín, 2007; Lago-Peñas and Dellal, 2010). On the one hand, external parameters like match venue (home/away), congested fixtures, or the respective league can affect match performance components (Rampinini et al., 2007; Dellal et al., 2011; Dolci et al., 2020). On the other hand, individual characteristics like anthropometry or physical capacities influence the physical, technical, and tactical output of players on the pitch (Aquino et al., 2020). Moreover, the influence of tactical factors on match performance has increasingly moved into the focus of scientific soccer research (Forcher et al., 2022e).

Tactical factors can be defined as variables affecting the convenient behavior of players to achieve the goals of the match (e.g., scoring goals). In detail, structural and functional tactical factors can be distinguished. On the one hand, structural tactical factors relate to the tactical positioning of a player in a playing position in a specific formation. On the other hand, functional tactical factors relate to the design of the playing environment in the context of a particular strategy. Typical structural tactical factors influencing match performance are the playing position of a player or the tactical formation (i.e., distribution of the 11 field players on the pitch) of a team (Bialkowski et al., 2014; Bauer et al., 2023). Regarding the influence of tactical factors, for example, wide players (i.e., wide defenders & wide midfielders) have been shown to exhibit more accelerations and greater sprinting distances than other playing positions (e.g., forwards) (Bush et al., 2015; Vigh-Larsen et al., 2017; Altmann et al., 2021). Furthermore, teams with a 3–5-2 formation are more compact and, therefore, can put more pressure on the opposing team than in other formations (Memmert et al., 2019).

Besides playing position and tactical formation, another well-studied functional tactical factor in soccer is the playing style of a team (Fernandez-Navarro et al., 2016). The playing style describes the behavior of the players at a team level. In detail, which characteristic behavioral features a team reveals that are repeated in their occurrence over a longer period (Fernandez-Navarro et al., 2016). A distinction is made between offensive (i.e., own ball possession) and defensive (i.e., opposing ball possession) playing styles, as the goals and thus the actions of the players in the respective phase of the match differ considerably (Fernandez-Navarro et al., 2016).

On the one hand, in the defensive phase, the team tries to prevent the opposition from scoring and regaining ball control (Bangsbo and Peitersen, 2000; Wright et al., 2011). On the other hand, in offensive match phases teams try to control the ball through possession and eventually score goals. In detail, emphasizing either one or the other of the two offensive objectives leads to fundamentally different offensive playing styles also known as ball possession and counter-attacking style. Firstly, some teams attempt to control the match with their ball possession and consequently try to disrupt the well-organized defending team with a series of passes (= ball possession style) (Forcher et al., 2021). Control through passing was perfected by Pep Guardiola at FC Barcelona and has since become known as “tiki-taka.” Secondly, other teams try to score goals by benefiting from the disrupted defense directly after the ball regain (= counter-attacking style) (Kempe et al., 2014). A well-known advocate of the counter-attacking opportunity is Ralf Rangnik (Fritsch, 2016). After successes as a manager with TSG Hoffenheim and RB Leipzig, he had meanwhile also arrived at Manchester United. These two offensive playing styles are the most reported in the current literature (Bate, 1988; Garganta et al., 1997; Tenga and Larsen, 2003; Redwood-Brown, 2008; Tenga et al., 2010a,b,c; Ruiz-Ruiz et al., 2013; Travassos et al., 2013). Both playing styles can be described as extremes on an offensive playing style continuum, which categorizes the offensive possession phases. Since previous research has focused mainly on offensive playing styles, this study will also examine offensive playing styles.

Already existing studies have mainly focused on the distinction and definition of offensive playing styles by analyzing performance data (Tenga and Larsen, 2003; Kempe et al., 2014). Since several studies have already shown the considerable influence of tactical factors on match performance, it seems worthwhile to also examine the playing style for its influence on match performance (Forcher et al., 2022e). A study by Yi et al. has already investigated the influence of offensive playing style on physical and technical match performance (Yi et al., 2019). However, only a small sample of 59 games was examined in this study. Furthermore, the investigation of Yi et al. did not consider that a team’s playing style can also change between several matches. Therefore, this study aimed to investigate the effect of offensive playing style (i.e., while in ball possession) on physical and technical match performance during offensive play as well as success-related factors. Since other tactical factors like the playing position or the tactical formation have been shown to influence physical and technical match performance (Forcher et al., 2022e), we hypothesized that the offensive playing style affects physical and technical match performance in professional soccer.

## 2. Materials and methods

### 2.1. Sample

In the present study, 153 matches of the 2020/21 German Bundesliga season were analyzed. The data basis was official tracking and event data. Due to existing restrictions regarding the accessibility of the match data, only the second half of the season could be analyzed in this study. The tracking data consisted of X and Y data of all 22 players on the pitch and the ball and were recorded by a semi-automated optical tracking system (TRACAB, ChyronHego, Melville, NY, United States). This system was recently considered valid [validity (spatial precision of position measurement): 0.07–0.18 m RMSE (root mean square errors); between device reliability of total distance: −0.15 m ± 0.37%] (Linke et al., 2020). The event data were raised manually by Sportec Solutions (Sportec Solutions AG, Ismaning, Germany) and the definitions of the events were based on an official checklist (Deutsche Fußball Liga (DFL), 2019). Tracking and event data were synchronized by matching the respective time-point of the tracking data for every event using the algorithm of Forcher et al. (2022b). All data processing and analysis were executed using Python 3.9 with the NumPy, Pandas, and Matplotlib libraries.

The study was conducted according to the guidelines of the Declaration of Helsinki and approved by the local ethics committee (Human and Business Sciences Institute, Saarland University, Germany, identification number: 22–02, 10 January 2022).

### 2.2. Procedures

To quantify the offensive playing style (i.e., ball possession or counter-attacking style) of each team in every match, we used and further developed a playing-style formula of Kempe et al. (2014). Therefore, we conducted a principle component analysis to weigh the physical and technical parameters within this already existing formula. This procedure allows a weighting of the parameters according to their importance concerning the classification into an offensive style of play. This new weighted formula is subsequently referred to as the playing style coefficient [PSC]. The PSC elevates the offensive style of play on an offensive playing style continuum between *ball possession* and *counter-attacking* style. While high PSC values are associated with a focus on ball possession, low PSC values are associated with a focus on counter-attacking.

The study by Yi et al. revealed that the offensive playing style significantly influences the ball possession rate of a team (Yi et al., 2019). Since the ball possession rate influences match performance (e.g., high-intensity running profile, number of passes), the present study examines all dependent variables solely during the ball possession of the respective team (Bradley et al., 2013; Mota et al., 2015). Therefore, similar to Goes et al. (2018) and Forcher et al. (2022b), ball possessions were defined as a phase where one team is controlling the ball. A possession ended with either the opponent gaining ball control or a stoppage of play (i.e., foul, offside, goal, final whistle, ball out of bounds). The dependent variables were examined at a team level and were categorized into three performance parts [physical (accelerations, decelerations, high-intensity distance, sprinting distance, and all four physical variables in relation to attacking time), technical (percentage of short passes, percentage of medium passes, percentage of long passes, percentage of horizontal passes, percentage of backward passes, dribblings, the success rate of all six technical parameters, and passing velocity), and success (xGoals, goals, and points)].

Firstly, the physical variables acceleration, deceleration, high-intensity distance, and sprint distance per attack were collected. Similar to Rhodes et al. predetermined thresholds for accelerations (> 3 m/s^2^) and decelerations (< −3 m/s^2^) were used (Rhodes et al., 2021). The high-intensity distance was defined as the distance where running speeds between 19.8–25.0 km/h are reached and the sprint distance with speeds above 25.0 km/h (Bradley et al., 2011; Tierney et al., 2016; Aquino et al., 2019; Borghi et al., 2020; Arjol-Serrano et al., 2021). Moreover, since it can be assumed that ball possession-oriented teams have longer ball possessions per attack, all physical variables were not only used as absolute values but also normalized based on attacking time and subsequently included as additional parameters.

Secondly, the technical variables passes and dribblings were raised. Additionally, for each technical parameter, the success rate was determined. Dribbling was recorded if a player in safe ball control tried to dribble past an opponent. Dribblings were considered successful if the respective player managed to dribble past the opponent. Furthermore, based on their distance, passes were categorized into short (<10 m), medium (10-30 m), and long (>30 m) (Forcher et al., 2022d). In addition, passes were classified backward or horizontal according to their playing angle (see Supplementary Figure S1). Since the results of Yi et al. suggest that ball possession oriented teams play more passes per attack, all passes were analyzed in relation to the total number of passes (Yi et al., 2019). Moreover, the average velocity of a pass was quantified. Passes were rated as successful when the ball reached a teammate.

Thirdly, as success-related variables points per match (0 = loss, 1 = draw, 3 = win), goals scored, and expected goals [xGoals] were recorded. xGoals were estimated after the definition of the German football league (Deutsche Fußball Liga (DFL), 2019).

Furthermore, the offensive tactical formation was captured by deploying the formation description algorithm by Forcher et al. (2022b). It clusters the average positions of all players into three formation lines (e.g., 4–4-2). The offensive formation represents the tactical distribution of all players on the pitch and is only measured for the team in possession (Forcher et al., 2022c).

$$\begin{array}{l} \mathrm{PSC}\;\left(playingstylecoefficient\right)=\left({\mathrm{PA}}^{*}\;0.35\right)\\ \;\;\;\;+\left({\mathrm{FP}}^{*}-0.03\right)+\left({\mathrm{TP}}^{*}\;0.23\right)+\;\left({\mathrm{PS}}^{*}\;0.32\right)\\ \;\;\;\;+\;\left({\mathrm{FPS}}^{*}\;0.32\right)+\left({\mathrm{BP}}^{*}\;0.32\right)+\left({\mathrm{DPA}}^{*}\;0.34\right)\\ \;\;\;\;+\left({\mathrm{RAT}}^{*}-0.32\right)+\;\left({\mathrm{MAT}}^{*}\;0.35\right)+\left({\mathrm{RD}}^{*}-0.24\right)+\left({\mathrm{MPA}}^{*}\;0.35\right). \end{array}$$

*PA ≙ Number of passes of one offensive action.*

*FP ≙ Number of passes forward in relation to the overall number of passes subtracted from 1.*

*TP ≙ Number of passes to a target player in relation to number of overall and non-target player passes.*

*PS ≙ Number of successful passes in relation to the overall number of passes.*

*FPS ≙ Number of successful passes forward in relation to the overall number of passes forward.*

*BP ≙ Sum of all periods of possession of one team in relation to the sum of the periods of possession of both teams.*

*DPA ≙ Distance covered during all attacks in relation to the total number of attacks.*

*RAT ≙ Mean time of the attack of the opponent subtracted by the own mean time of the attack.*

*MAT ≙ Relation of the total time of all attacks to the number of attacks.*

*RD ≙ Relation of the distance covered within one attack to the time with ball possession.*

*MPA ≙ Relation of the total number of passes to the total number of attacks.*

### 2.3. Evaluation of the playing style coefficient

Since the PSC is a new formula, it was examined *a priori* for its validity. To evaluate the PSC, the results of the *PSC for every match performance*, the results of the *formula of* Kempe et al. (2014), and the results of a *formula based on an expert rating* were compared. The *formula based on an expert rating* was developed by weighting the individual parameters based on the rating of three licensed and experienced coaches of a professional club. All three raters independently rated the parameters according to their importance for the quantification of the offensive playing style with the help of a questionnaire (i.e., each variable could be classified as important, neutral, or unimportant). To compare the results of the three calculations, all 18 included teams were sorted in a table (i.e., from ball possession to counter-attacking focused) based on their average values (i.e., average score over all 17 matches). Before executing the three alternating calculations, the values were transformed into z-scores. To compare the table results of the three calculations, a Spearman rank correlation was calculated between the tables based on the results of all three formulas (see Supplementary Table S1). As the results between the *PSC*, the previously evaluated *formula by* Kempe et al., and the *formula based on the expert rating* showed a high degree of agreement (*ρ* = 0.93–0.97; 95% CI = 0.77–0.99; *p* < 0.01), the *PSC* was assessed as valid.

### 2.4. Statistical analysis

For each dependent physical (e.g., sprinting distance), technical (e.g., dribblings), and success (e.g., xGoals) variable a single repeated measures linear mixed model was conducted using the statsmodels library in Python 3.9. The value of the PSC served as the fixed effect for each model. Hence, the PSC is the independent variable used to predict the respective dependent variable (i.e., physical, technical, or success variable). Each physical variable was examined in absolute form and in relation to the attacking time.

A hierarchical modeling strategy was implemented, following the example of Fernandez-Navarro et al. (2018). Therefore, random effects (i.e., team, offensive formation) were added step by step for each model independently. Hence, depending on the model, a different number of random effects were the consequence. The data structure was hierarchical, as, for example, all teams are ranked higher than one single team (Heck et al., 2014).

To evaluate the model performance, the Akaike criterion [AIC] was used (i.e., lower AIC values = better model). Furthermore, restricted maximum likelihood (REML) estimation was implemented for model fitting. The statistical significance level was set *a priori* at *p* < 0.05.

## 3. Results

**Figure 1:**
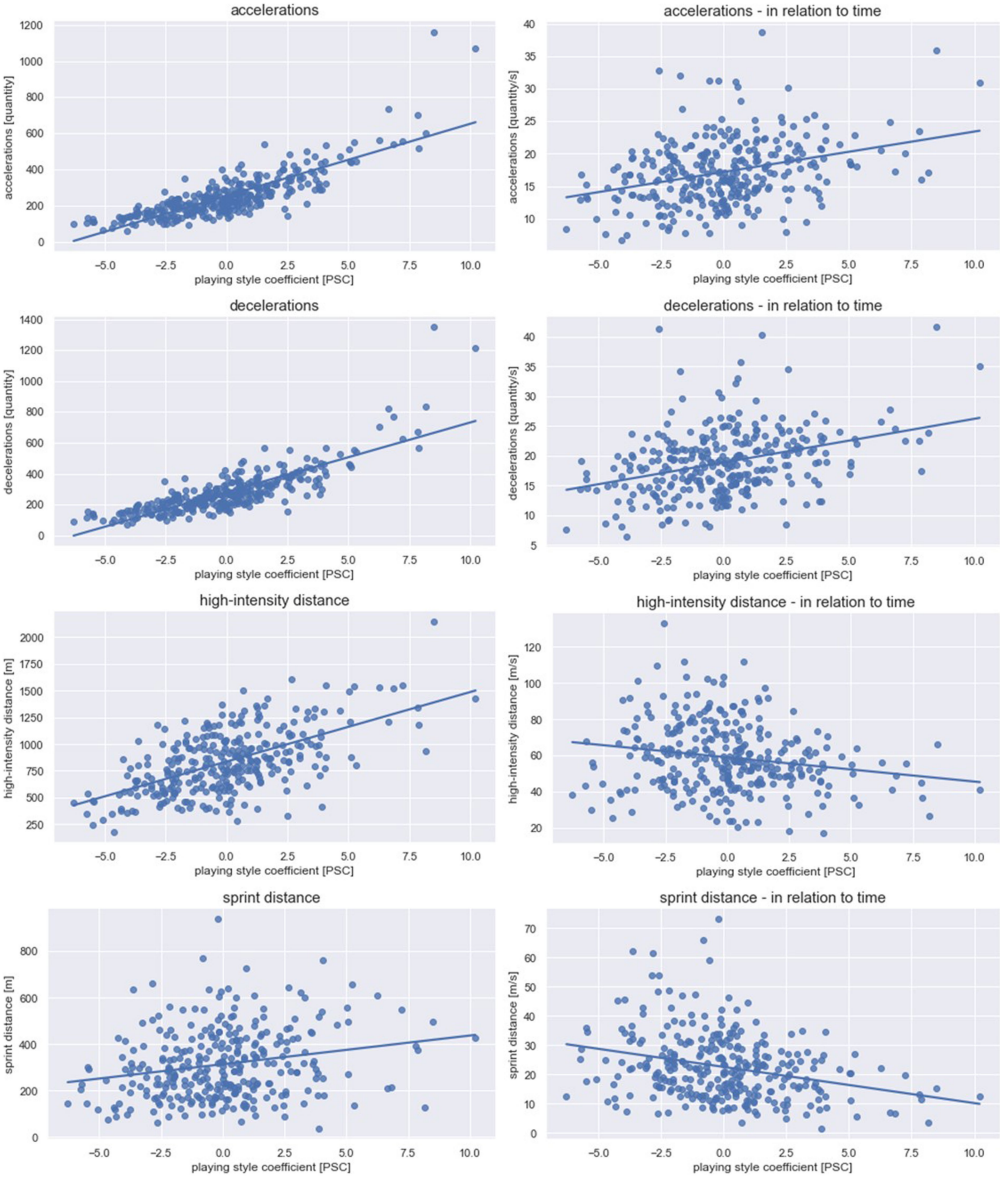
**Physical match performance.** Data for the physical parameters are presented. One data point depicts one team in one match. The line represents the linear regression between the playing style coefficient [PSC] and the dependent physical variable. While high PSC values indicate a ball possession focus, low PSC values indicate a counter-attack focus.

**Figure 2:**
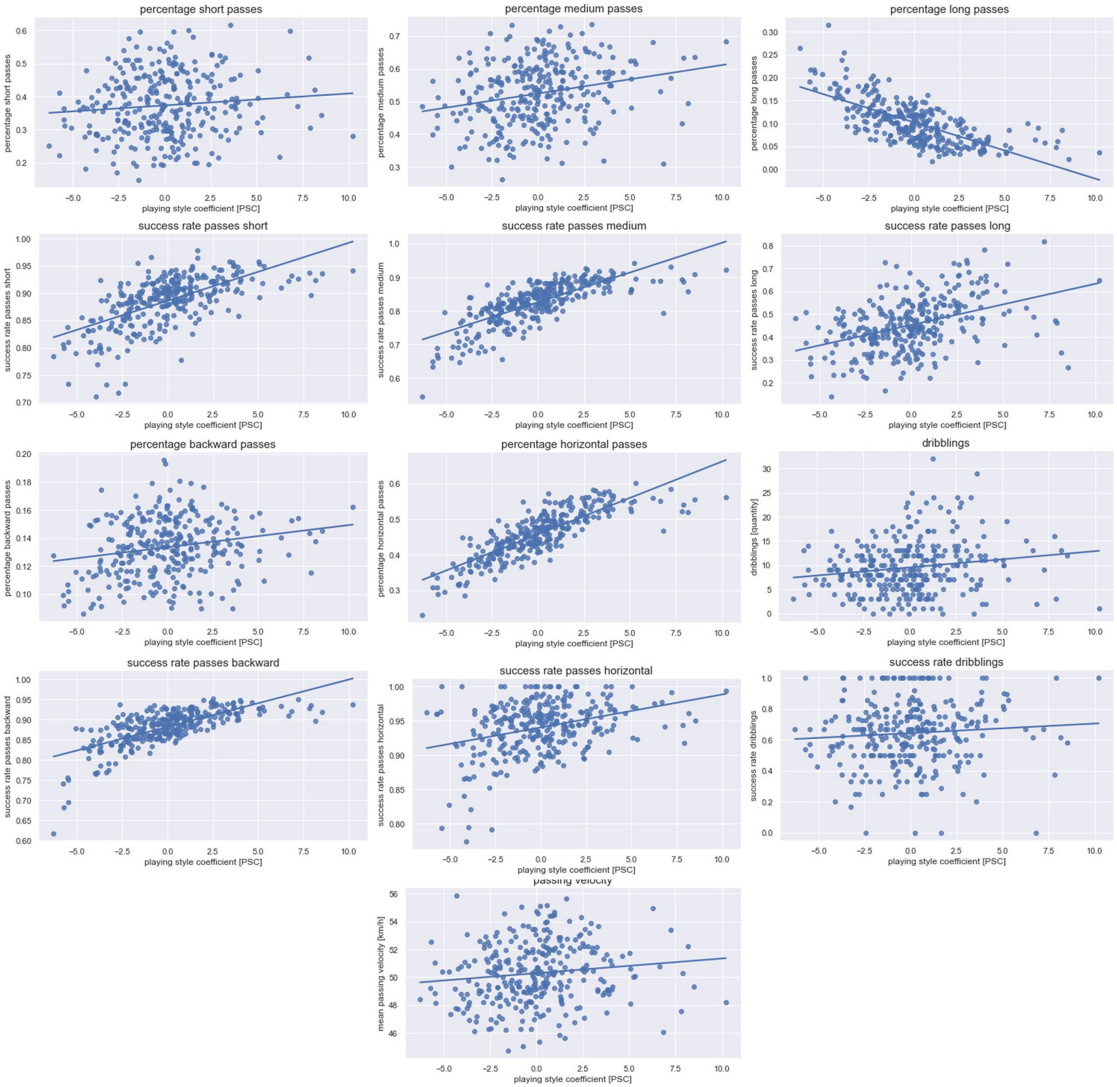
**Technical match performance.** Data for the technical parameters are presented. One data point depicts one team in one match. The line represents the linear regression between the playing style coefficient [PSC] and the dependent technical variable. While high PSC values indicate a ball possession focus, low PSC values indicate a counter-attack focus.

**Figure 3:**
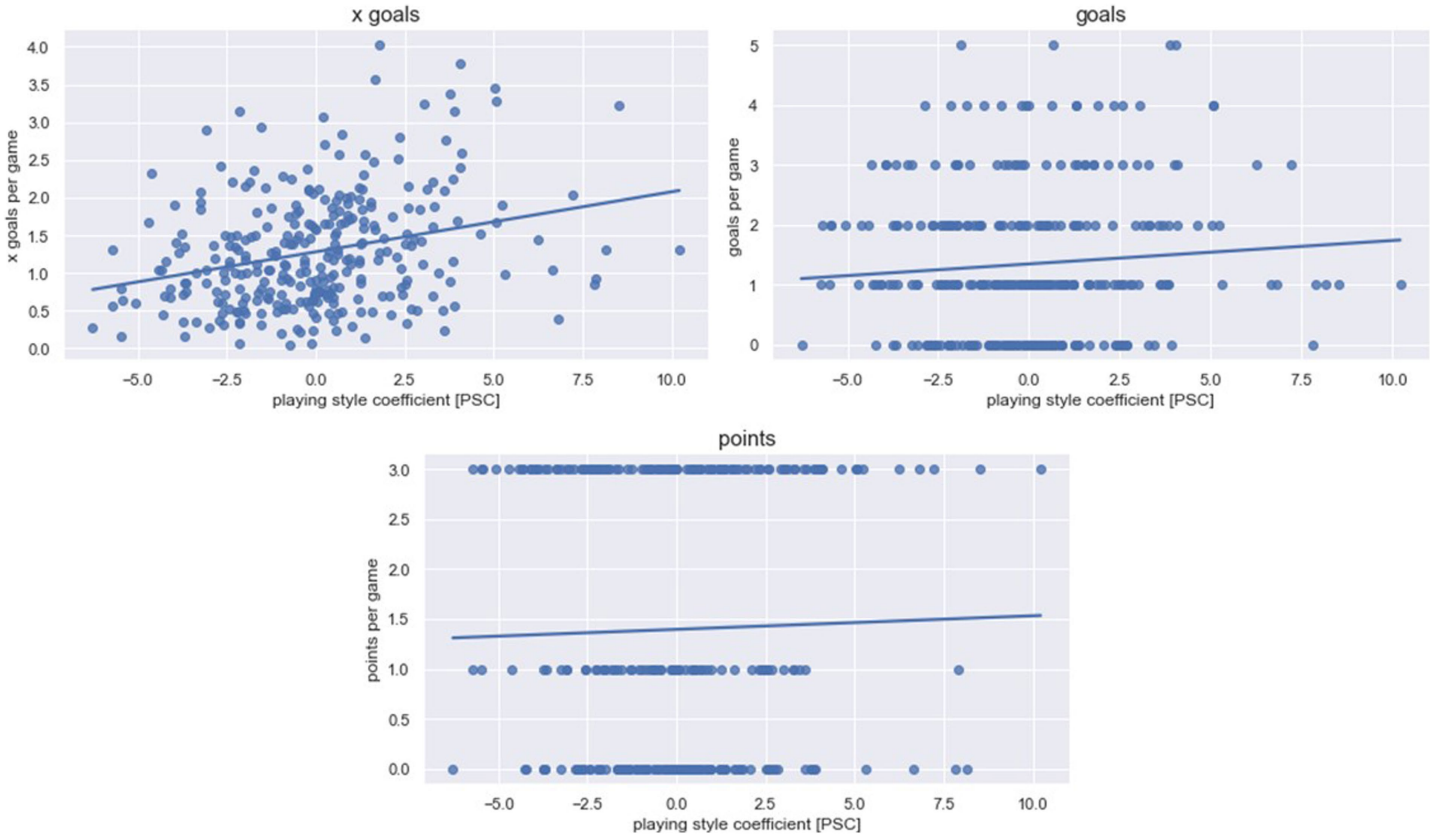
**Success.** Data for the success factors are presented. One data point depicts one team in one match. The line represents the linear regression between the playing style coefficient [PSC] and the dependent success variable. While high PSC values indicate a ball possession focus, low PSC values indicate a counter-attack focus.

A total of 9,546 attacks were evaluated [Ø = 31.2 attacks per team per match, standard deviation (SD) = 8.4], of which one attack lasted on average 14.65 s (SD = 4.62). The average PSC value was 0.00 with values ranging from a minimum of −6.27 to a maximum of 10.21 for one team in a single match. Figures 1–3 illustrate the results concerning the influence of the PSC on the respective dependent variable graphically. Table 1 provides detailed information on each linear mixed model including the weights of effects. The random effect *team membership* improved each model. The random effect *offensive formation* improved the model only for selected parameters and was therefore excluded for all other parameters. Additional information on the means and SD for the variables used in the PSC formula and the dependent variables can be found in Supplementary Tables S2–S4.

**Table 1:** Linear mixed models.

linear mixed models (LMM)	*β*	SE	95% CI		*z*	*p*
Physical match performance						
Accelerations						
Fixed effect						
Intercept	257.73	7.03	243.96	271.50	36.69	<0.01
Playing style coefficient [PSC]	38.75	1.75	35.33	42.18	22.17	<0.01
Random effects						
Teams	1032.09	9.42				
Offensive formation	6.47	0.17				
*R* ^2^	0.69					
Accelerations in relation to time						
Fixed effect						
Intercept	17.21	0.58	16.07	18.35	29.54	<0.01
Playing style coefficient [PSC]	0.33	1.16	0.10	0.56	2.86	<0.01
Random effects						
Teams	6.52	0.79				
Offensive formation	0.04	0.01				
*R* ^2^	0.23					
Decelerations						
Fixed effect						
Intercept	284.26	7.77	269.03	299.50	36.58	<0.01
Playing style coefficient [PSC]	45.45	1.95	41.63	49.27	23.33	<0.01
Random effects						
Teams	1434.12	11.10				
Offensive formation	13.216	0.23				
*R* ^2^	0.69					
Decelerations in relation to time						
Fixed effect						
Intercept	18.86	0.57	17.74	19.99	32.89	<0.01
Playing style coefficient [PSC]	0.51	0.13	0.26	0.76	4.00	<0.01
Random effects						
Teams	−0.36	0.73				
Offensive formation	0.11	0.03				
R^2^	0.22					
Sprinting distance						
Fixed effect						
Intercept	313.50	12.44	289.12	337.89	25.20	<0.01
Playing style coefficient [PSC]	7.87	3.72	0.58	15.17	2.11	0.03
Random effects						
Teams	1615.47	7.67				
Offensive formation	Not included					
R^2^	0.08					
Sprinting distance in relation to time						
Fixed effect						
Intercept	22.60	1.06	20.53	24.67	21.36	<0.01
Playing style coefficient [PSC]	−1.72	0.28	−2.27	−1.18	−6.17	<0.01
Random effects						
Physical match performance						
teams	13.77	0.75				
offensive formation	Not included					
*R* ^2^	0.14					
High-intensity distance						
Fixed effect						
Intercept	835.57	19.40	797.54	873.60	43.07	<0.01
Playing style coefficient [PSC]	60.92	5.75	49.65	72.18	10.60	<0.01
Random effects						
Teams	3619.64	10.78				
Offensive formation	Not included					
*R* ^2^	0.36					
High-intensity distance in relation to time						
Fixed effect						
Intercept	58.89	2.00	54.96	62.81	29.39	<0.01
Playing style coefficient [PSC]	−2.19	0.45	−3.08	−1.30	−4.82	<0.01
Random effects						
Teams	54.51	1.54				
Offensive formation	Not included					
*R* ^2^	0.13					

Linear mixed models (LMM)						
Technical match performance						
Percentage short passes						
Fixed effect						
Intercept	0.37	32.72	0.35	0.40	32.72	<0.01
Playing style coefficient [PSC]	0.00	0.00	0.46	0.00	0.74	0.46
Random effects						
Teams	0.00	0.01				
Offensive formation	Not included					
*R* ^2^	0.17					
Success rate passes short						
Fixed effect						
Intercept	0.89	0.00	0.88	0.89	223.42	<0.01
Playing style coefficient [PSC]	0.01	0.00	0.01	0.01	12.03	<0.01
Random effects						
Teams	0.00	0.00				
Offensive formation	Not included					
*R* ^2^	0.46					
Percentage medium passes						
Fixed effect						
Intercept	0.53	0.01	0.52	0.55	60.00	<0.01
Playing style coefficient [PSC]	0.01	0.00	0.00	0.01	3.70	<0.01
Random effects						
Teams	0.00	0.01				
Offensive formation	0.01	0.01				
*R* ^2^	0.20					
Technical match performance						
Success rate passes medium						
Fixed effect						
Intercept	0.83	0.00	0.82	0.84	225.88	<0.01
Playing style coefficient [PSC]	0.02	0.00	0.01	0.02	15.71	<0.01
Random effects						
Teams	0.00	0.00				
Offensive formation	0.00	0.00				
*R* ^2^	0.62					
Percentage long passes						
Fixed effect						
Intercept	0.10	0.00	0.10	0.11	24.78	<0.01
Playing style coefficient [PSC]	−0.01	0.00	−0.01	−0.01	−12.74	<0.01
Random effects						
teams	0.00	0.00				
offensive formation	0.00	0.00				
*R* ^2^	0.58					
Success rate passes long						
Fixed effect						
Intercept	0.45	0.01	0.43	0.48	37.32	<0.01
Playing style coefficient [PSC]	0.01	0.00	0.01	0.02	5.67	<0.01
Random effects						
Teams	0.00	0.01				
Offensive formation	Not included					
*R* ^2^	0.29					
Percentage horizontal passes						
Fixed effect						
Intercept	0.46	0.00	0.45	0.47	123.60	<0.01
Playing style coefficient [PSC]	0.02	0.00	0.02	0.02	20.77	<0.01
Random effects						
Teams	0.00	0.00				
Offensive formation	0.00	0.01				
*R* ^2^	0.73					
Success rate passes horizontally						
Fixed effect						
Intercept	0.94	0.00	0.93	0.95	258.19	<0.01
Playing style coefficient [PSC]	0.01	0.00	0.00	0.01	5.30	<0.01
Random effects						
Teams	0.00	0.00				
Offensive formation	0.00	0.01				
*R* ^2^	0.17					
Percentage backward passes						
Fixed effect						
Intercept	0.13	0.00	0.13	0.14	61.57	<0.01
Playing style coefficient [PSC]	0.00	0.00	0.00	0.00	2.65	<0.01
Technical match performance						
Random effects						
Teams	0.00	0.00				
Offensive formation	Not included					
*R* ^2^	0.15					
Success rate passes backward						
Fixed effect						
Intercept	0.88	0.00	0.88	0.89	328.75	<0.01
Playing style coefficient [PSC]	0.01	0.00	0.01	0.01	15.79	<0.01
Random effects						
Teams	0.00	0.00				
Offensive formation	Not included					
*R* ^2^	0.51					
Passing velocity						
Fixed effect						
Intercept	50.29	0.35	49.61	50.98	144.03	<0.01
Playing style coefficient [PSC]	0.21	0.04	0.13	0.30	4.81	<0.01
Random effects						
Teams	2.02	0.46				
Offensive formation	Not included					
*R* ^2^	0.37					
Dribblings						
Fixed effect						
Intercept	9.55	0.57	8.43	10.67	16.75	<0.01
Playing style coefficient [PSC]	0.19	0.13	−0.05	0.44	1.55	0.12
Random effects						
Teams	4.42	0.43				
Offensive formation	Not included					
*R* ^2^	0.13					
Success rate dribblings						
Fixed effect						
Intercept	0.66	0.02	0.61	0.70	29.25	<0.01
Playing style coefficient [PSC]	0.00	0.01	−0.01	0.01	−0.06	0.95
Random effects						
Teams	0.00					
Offensive formation	0.04					
*R* ^2^	0.07					

Linear mixed models (LMM)						
Success related factors						
*X* Goals						
Fixed effect						
Intercept	1.28	0.07	1.15	1.41	19.57	<0.01
Playing style coefficient [PSC]	0.06	0.02	0.02	0.09	3.19	<0.01
Random effects						
Teams	0.05	0.04				
Offensive formation	Not included					
Success related factors						
*R* ^2^	0.13					
Goals						
Fixed effect						
Intercept	1.35	0.15	1.06	1.64	9.14	<0.01
Playing style coefficient [PSC]	−0.05	0.03	−0.10	0.01	−1.49	0.14
Random effects						
Teams	0.32	0.13				
Offensive formation						
*R* ^2^	0.13					
Points						
Fixed effect						
Intercept	1.37	0.14	1.10	1.64	9.86	<0.01
Playing style coefficient [PSC]	−0.03	0.04	−0.10	0.04	−0.92	0.36
Random effects						
Teams	0.11	0.10				
Offensive formation	1.54	0.68				
*R* ^2^	0.10					

Results of the linear mixed models with the physical, technical, and success parameters as dependent variable. The coefficients of the effects (*β*), the standard error (SE), the 95% confidence interval and the *z*-and according *p*-values are presented. The fixed effect playing style coefficient [PSC] and the random effects team and offensive formation are distinguished. If a random effect was excluded the row was labelled with ‘not included’.

For all physical variables, the influence of the PSC was significant (*p* < 0.03). High *R*^2^ values were found for accelerations (*R*^2^ = 0.69; *β* = 38.75; *p* < 0.01) and decelerations (*R*^2^ = 0.69; *β* = 45.45; *p* < 0.01). Lower *R*^2^ was found for sprinting distance (*R*^2^ = 0.08; *β* = 7.87; *p* = 0.03), high-intensity distance (*R*^2^ = 0.36; *β* = 60.92; *p* < 0.01), accelerations in relation to time (*R*^2^ = 0.23; *β* = 0.33; *p* < 0.01), decelerations in relation to time (*R*^2^ = 0.22; *β* = 0.51; *p* < 0.01), sprinting distance in relation to time (*R*^2^ = 0.14; *β* = −1.72; *p* < 0.01), and high-intensity distance in relation to time (*R*^2^ = 0.13; *β* = −2.19; *p* < 0.01).

The influence of the PSC was significant for all technical variables (*p* < 0.01), except for the percentage short passes, dribblings, and success rate of dribblings (*p* = 0.12–0.95). High values for R^2^ can be found for the parameters percentage long passes (*R*^2^ = 0.58; *β* = −0.01; *p* < 0.01), percentage horizontal passes (*R*^2^ = 0.73; *β* = 0.02; *p* < 0.01) as well as the success rate of short (R^2^ = 0.46; *β* = 0.01; *p* < 0.01), medium (*R*^2^ = 0.62; *β* = 0.02; *p* < 0.01), and backward (*R*^2^ = 0.51; *β* = 0.01; *p* < 0.01) passes. Lower *R*^2^ values were revealed for the parameters percentage short passes (*R*^2^ = 0.17; *β* = 0.00; *p* = 0.46), percentage medium passes (*R*^2^ = 0.20; *β* = 0.01; *p* < 0.01), percentage backward passes (*R*^2^ = 0.15; *β* = 0.00; *p* < 0.01), passing velocity (*R*^2^ = 0.37; *β* = 0.21; *p* < 0.01), and dribblings (R^2^ = 0.13; *β* = 0.19; *p* = 0.12), as well as the success rate of dribblings (*R*^2^ = 0.07; *β* = 0.00; *p* = 0.95), long (*R*^2^ = 0.29; *β* = 0.01; *p* < 0.01), and horizontal (*R*^2^ = 0.17; *β* = 0.01; *p* < 0.01) passes.

Concerning the success parameters, the influence of the PSC was solely significant for xGoals (*R*^2^ = 0.13; *β* = 0.06; *p* < 0.01). It was not significant for goals (*R*^2^ = 0.13; *β* = −0.05; *p* = 0.14) and points (*R*^2^ = 0.10; *β* = −0.03; *p* = 0.36).

## 4. Discussion

The present study revealed an effect of the offensive playing style (i.e., while in ball possession) on physical and technical match performance during offensive play in professional soccer. However, the influence of the offensive playing style on success-related variables was marginal. In detail, teams with a ball possession style were more physically demanded over a whole match (e.g., more accelerations/decelerations, high-intensity, sprinting distance). In contrast, teams with a counter-attacking style covered more high-intensity and sprinting distance normalized at the attacking time. Furthermore, on the one hand, teams with a ball possession style played more horizontal passes and had better passing success rates. On the other hand, counter-attacking style teams played more long passes.

To gain a better understanding of the influence of the offensive playing style on match performance, the results and discussion of the physical match performance, technical match performance, and success-related variables will be considered separately.

### 4.1. Physical match performance

All physical match performance parameters examined in this study were significantly influenced by the offensive style of play. In detail, with increasing PSC values (i.e., emphasis on ball possession) the number of accelerations and decelerations, as well as the distance in high-intensity and sprinting speeds per match increased. Moreover, Yi et al. found similar results, indicating higher high-intensity and sprinting distances for ball-possession style teams (Yi et al., 2019). However, it should be noted that Yi et al. investigated the physical match performance of a whole match (i.e., also during opposing ball possession). Accordingly, the offensive playing style of ball possession is associated with an additional physical effort for the players. It is important to highlight, that since physical match performance increases with effective playing time (Altmann et al., 2023), it can be assumed that the increased attacking time regarding the ball possession style (see Supplementary Table S3, e.g., team 17) is the reason for the higher physical match performance of ball possession-oriented teams.

Furthermore, high-intensity and sprint distances decreased with increasing PSC value (i.e., emphasis on ball possession style), when analyzing the distances normalized at the attacking time. One could conclude that after gaining the ball, teams with an emphasis on counter-attacking style have to cover a large distance at high speeds in transition to get in front of the opponent’s goal. This has to happen as quickly as possible to use the short time when the opposing defense is disorganized. In contrast, teams with a focus on ball possession have more time since they face an orderly opponent and try to disorganize him with several successive passes. Therefore, the distance to the opponent’s goal can be covered with lower speeds. However, the number of accelerations and decelerations normalized by attacking time remains the same comparing both ends of the playing style continuum. This relationship reveals that irrespective of the offensive playing style, short high-intensity actions (e.g., accelerations and decelerations) are necessary to get in goal-threatening situations.

### 4.2. Technical match performance

In terms of the technical match performance, the offensive playing style influenced the technical parameters to varying degrees. On the one hand, the percentage of short passes, the percentage of medium passes, the average passing velocity, the number of dribblings, and the success rate of dribblings are influenced by the offensive playing style to only a small extent. Accordingly, it can be stated that teams of both extreme ends on the playing style continuum play a similarly high percentage of short and medium passes. One possible explanation for this finding is that short and medium passes are used very frequently regardless of the style of play (see Supplementary Table S4). Both styles of play also go along with a similar amount of dribblings. There seems to be no difference in the amount of dribbling, as both playing styles use dribblings only to a small extent (see Supplementary Table S4).

On the other hand, the percentage of long passes is strongly influenced by the offensive playing style. With a growing focus on counter-attacking (=PSC values decreased), the proportion of long passes increased. As explained above, counter-attacking teams try to quickly bridge the space to the opponent’s goal in transition play. To optimally achieve this objective, counter-attacking teams play more long passes. In contrast, ball possession teams try to control the ball throughout longer periods (see Supplementary Table S3, e.g., team 1). Since long passes increase the risk of losing the ball (see passing success rates), ball possession-style teams play fewer long passes to reduce the risk of losing ball control.

The abovementioned conclusion is also supported by the results for the passing success rates. For almost all passes (e.g., medium, backward), teams with a focus on ball possession revealed better success rates than counter-attacking style teams. This finding is supported by the results of Yi et al., who similarly found better passing success for ball-possession style teams (Yi et al., 2019). As already explained, a poorer passing rate leads to more ball losses and consequently less control of the match. Consequently, ball possession-oriented teams need to have high-quality passing success rates to control the match by possession and thus allow little possession time for the opponent.

Moreover, with a rising focus on a ball possession style (= higher PSC values), the percentage of horizontal passes increased. As counter-attacking style teams try to exploit the disorganized opponent directly after gaining ball control, they have to cover the long distance to the opponent’s goal with not only long but also vertical passes (see Supplementary Table S3, e.g., team 15). Teams with a focus on ball possession pursue the approach of destabilizing a defensively organized opponent through targeted passing (Forcher et al., 2021). Therefore, a larger percentage of the passes needs to be played horizontally, for example, to enable lateral shifts to destabilize the opponent and hence receive scoring opportunities.

### 4.3. Success

In contrast to physical and technical match performance, the influence of offensive playing style on success-related parameters remained small. Concluding, values that are strongly influenced by chance, such as goals and points (Brechot and Flepp, 2020) (i.e., points awarded for the match outcome, e.g., three, two, or zero), are not influenced by the style of play. In contrast, findings by Yi et al. suggested that a focus on a ball-possession style is associated with an increased probability of success (Yi et al., 2019). In the context of the present study, the only success-related parameter significantly interacting with the PSC was xGoals. In detail, there was a slight tendency for teams with a greater focus on ball possession to achieve more xGoals than teams with an accent on counter-attacking. A possible explanation for this result could be related to the two different playing styles and their objectives. A common observation in professional soccer suggests a higher focus on defensive play for teams playing against a stronger opponent. Therefore, these weaker teams (i.e., in relation to the opponent) strongly focus on the objective of not conceding a goal. Consequently, their offensive play is limited to a few counter-attacking opportunities, which decreases the chance of realizing a large number of scoring opportunities (i.e., potentially low xGoals value). The opposite scenario can be observed with teams playing against a weaker opponent (i.e., focus on offensive play potentially leads to more scoring opportunities). Thus, these stronger teams (i.e., in relation to the opponent) could be associated with ball-possession and weaker teams with a counter-attacking style which possibly leads to the observed difference in xGoals (Kempe et al., 2014).

### 4.4. Limitations and future research

To obtain a complete picture of the present study, the limitations should be considered in the following. Since the used data is from the Bundesliga and the dependence of match performance on country and league is confirmed, the transfer of results and conclusions is limited (Rampinini et al., 2007; Dellal et al., 2011). In addition, only a continuum of two offensive playing styles was considered in the survey of different styles of play (Fernandez-Navarro et al., 2016). This may represent a simplification of reality. Furthermore, to strengthen the significance of the findings in this study an already validated formula was used to determine the style of play. However, in this calculation, various technical and physical parameters were included and, therefore, it cannot be precluded that those variables are independent of the dependent physical, technical, and success variables used in this study. Moreover, the opponent was not considered in the present study. Since performance in soccer arises from the interaction between the two teams, the opponent should be considered in future studies. This leads directly to the topics for future research.

Fruitful avenues for future studies could be to examine the parameters collected on an individual level and, for example, consider other contextual factors influencing soccer match performance (e.g., playing position, quality of the teams) (Forcher et al., 2022d). Furthermore, an investigation of the influence of defensive playing style on match performance during defensive play could complete the picture of the current study (Forcher et al., 2022a).

## 5. Conclusion

This is one of the first studies examining the influence of offensive playing style on soccer match performance and, therefore, enhances our understanding regarding performance characteristics of different offensive playing styles. The offensive playing style influences the technical and physical match performance considerably, with success-related variables only being affected to a small extent.

While counter-attacking style teams covered more high-intensity and sprint distances normalized at the attacking time, teams with a focus on ball possession were physically more demanded in consideration of a whole match (e.g., accelerations, decelerations, high-intensity, sprint distances). Furthermore, ball possession-oriented teams played more horizontal passes and revealed better passing success rates. In contrast, counter-attacking teams played more long passes.

The findings are particularly relevant for coaches and practitioners working in professional soccer clubs, who can use the findings to better interpret physical and technical match performance data. In detail, insights can be used to draw conclusions about changes in match performance (e.g., influence of playing style). Furthermore, training content can be optimized accordingly. Therefore, players can be trained for the demands (e.g., technical and physical match performance demands) that go along with the style of play that the coach wants his team to play. In addition, performance analysts can use the playing style coefficient to categorize teams on a continuum between ball-possession style and counter-attacking style. This can provide information that helps to characterize the upcoming opponent and prepare the own players for possible specialties of the opponent. However, since the effect of the offensive playing style on success-related factors is minor, coaches can still freely decide which offensive playing style does fit their philosophy and players without affecting the chance of success *per se*.

## Data availability statement

The data that support the findings of this study are available from the Deutsche Fußball Liga (DFL). Restrictions apply to the availability of these data, which were used under license for this study. Therefore, the data is not freely available. Requests to access these datasets should be directed to info@dfl.de.

## Ethics statement

The studies involving human participants were reviewed and approved by the Human and Business Sciences Institute, Saarland University, Germany, identification number: 22–02, 10 January 2022. Written informed consent for participation was not required for this study in accordance with the national legislation and the institutional requirements.

## Author contributions

LOF, SA, and TG: conceptualization. LOF and LAF: investigation. LOF and SA: methodology. HW, DJ, SA, and AW: supervision. LOF and TG: validation. LOF, SA, LAF, HW, DJ, AW, and TG: writing. All authors contributed to the article and approved the submitted version.

## Conflict of interest

The authors declare that the research was conducted in the absence of any commercial or financial relationships that could be construed as a potential conflict of interest.

## Publisher’s note

All claims expressed in this article are solely those of the authors and do not necessarily represent those of their affiliated organizations, or those of the publisher, the editors and the reviewers. Any product that may be evaluated in this article, or claim that may be made by its manufacturer, is not guaranteed or endorsed by the publisher.
