# Supplementary material for: Is ball-possession style more physically demanding than counter-attacking? The influence of playing style on match performance in professional soccer
Source: Front Psychol. 2023 Jul 7;14:1197039. doi: 10.3389/fpsyg.2023.1197039 (PMC10361297; doi:10.3389/fpsyg.2023.1197039)
Supplement: Supplementary file 1 [file Table_1.DOCX]

**Supplementary Table 1.** Spearman correlation between results based on the formula of Kempe et al. (2014), the formula based on the expert rating, and the formula based on the weighting according to the results of the principle component analysis [PCA]. Based on the results of the three alternating calculations teams were ranked on a continuum between counter-attacking- and possession-oriented. The table shows the rank correlation [ρ] between the results of the three different calculations, the significance value [p] and the 95% confidence interval [95% CI].

|  | **based on Kempe et al.** | **based on expert rating** | **based on PCA** |
| --- | --- | --- | --- |
| **based on Kempe et al.** |  | ρ=0.97; 95% CI=0.87-0.99; p<0.01 | ρ=0.97; 95% CI=0.88-0.99; p<0.01 |
| **based on expert rating** | ρ=0.97; 95% CI=0.87-0.99; p<0.01 |  | ρ=0.93; 95% CI=0.77-0.97; p<0.01 |
| **based on PCA** | ρ=0.97; 95% CI=0.88-0.99; p<0.01 | ρ=0.93; 95% CI=0.77-0.97; p<0.01 |  |
