# Supplementary material for: Is ball-possession style more physically demanding than counter-attacking? The influence of playing style on match performance in professional soccer
Source: Front Psychol. 2023 Jul 7;14:1197039. doi: 10.3389/fpsyg.2023.1197039 (PMC10361297; doi:10.3389/fpsyg.2023.1197039)
Supplement: Supplementary file 2 [file Table_2.DOCX]

# **Supplementary Table 2.** Means ± SD for parameters (z-transformed values) included in the formula for the playing style coefficient [PSC] for every team.

|  | **Passes per action** | | **Forward passing** | | **Target player passes** | | **Passing success rate** | | **Forward passing success rate** | | **Ball possession rate** | |
| --- | --- | --- | --- | --- | --- | --- | --- | --- | --- | --- | --- | --- |
|  | **mean** | **SD** | **mean** | **SD** | **mean** | **SD** | **mean** | **SD** | **mean** | **SD** | **mean** | **SD** |
| **team 1** | 1.11 | 1.16 | 0.02 | 0.85 | 0.56 | 0.55 | 0.80 | 0.77 | 0.84 | 0.83 | 0.54 | 0.87 |
| **team 2** | 0.45 | 1.04 | -0.10 | 1.37 | 0.50 | 0.75 | 0.38 | 0.90 | 0.42 | 0.93 | 0.16 | 0.97 |
| **team 3** | 0.35 | 0.45 | -0.27 | 0.86 | 0.72 | 0.54 | 0.80 | 0.48 | 0.70 | 0.59 | 0.57 | 1.00 |
| **team 4** | 0.68 | 1.65 | -0.04 | 0.82 | 0.39 | 0.37 | 0.63 | 0.56 | 0.56 | 0.62 | 0.86 | 0.87 |
| **team 5** | -0.35 | 0.88 | -0.05 | 1.12 | -0.13 | 1.08 | -0.18 | 0.70 | -0.26 | 0.72 | 0.21 | 0.89 |
| **team 6** | -0.10 | 0.55 | -0.34 | 0.88 | 0.11 | 0.54 | 0.00 | 0.77 | 0.05 | 0.81 | -0.29 | 0.67 |
| **team 7** | -0.07 | 0.67 | 0.61 | 0.75 | 0.51 | 0.78 | 0.34 | 0.85 | 0.44 | 0.93 | 0.29 | 0.70 |
| **team 8** | -0.20 | 0.70 | -0.70 | 1.03 | -0.46 | 0.83 | -0.14 | 0.78 | -0.28 | 0.89 | -0.24 | 0.93 |
| **team 9** | 0.13 | 1.66 | -0.09 | 0.79 | -0.31 | 0.81 | -0.14 | 0.67 | -0.31 | 0.64 | -0.38 | 1.00 |
| **team 10** | -0.25 | 0.58 | 0.11 | 1.12 | -0.02 | 0.79 | 0.08 | 0.73 | -0.04 | 0.91 | 0.06 | 0.91 |
| **team 11** | -0.38 | 0.57 | -0.15 | 0.86 | -0.48 | 1.18 | -1.10 | 1.16 | -1.08 | 1.06 | -0.86 | 0.91 |
| **team 12** | -0.40 | 0.45 | 0.09 | 0.93 | -0.27 | 0.91 | -0.25 | 0.83 | -0.29 | 0.88 | -0.49 | 0.82 |
| **team 13** | -0.29 | 0.52 | -0.36 | 0.88 | -0.62 | 1.25 | -0.37 | 0.72 | -0.33 | 0.64 | -0.31 | 0.84 |
| **team 14** | 0.07 | 1.08 | 0.56 | 1.15 | 0.44 | 0.56 | 0.30 | 0.54 | 0.36 | 0.59 | 0.18 | 0.94 |
| **team 15** | -0.85 | 0.55 | 0.98 | 0.90 | -1.00 | 1.35 | -1.43 | 1.04 | -1.19 | 0.91 | -0.72 | 0.92 |
| **team 16** | 0.04 | 0.58 | -0.29 | 0.96 | 0.29 | 0.65 | 0.40 | 0.68 | 0.33 | 0.71 | 0.21 | 0.79 |
| **team 17** | 0.79 | 1.02 | 0.14 | 0.65 | 0.74 | 0.57 | 0.95 | 0.51 | 1.04 | 0.59 | 1.07 | 0.65 |
| **team 18** | -0.73 | 0.45 | -0.12 | 0.96 | -0.97 | 1.23 | -1.06 | 0.93 | -0.96 | 0.94 | -0.87 | 0.74 |
|  | **Distance per attack** | | **Relative attacking time** | | **Mean attacking time** | | **Running distance in relation to the time of an attack** | | **Mean passes per attack** | | **PSC** | |
|  | **mean** | **SD** | **mean** | **SD** | **mean** | **SD** | **mean** | **SD** | **mean** | **SD** | **mean** | **SD** |
| **team 1** | 1.00 | 1.18 | -0.86 | 0.92 | 1.08 | 1.26 | -0.71 | 0.91 | 1.11 | 1.16 | 2.76 | 2.82 |
| **team 2** | 0.23 | 1.07 | -0.26 | 0.91 | 0.31 | 1.09 | -0.44 | 1.02 | 0.45 | 1.04 | 1.11 | 2.66 |
| **team 3** | 0.14 | 0.48 | -0.27 | 0.84 | 0.14 | 0.42 | -0.28 | 0.53 | 0.35 | 0.45 | 1.33 | 1.22 |
| **team 4** | 0.50 | 1.52 | -0.66 | 1.15 | 0.45 | 1.52 | -0.01 | 0.79 | 0.68 | 1.65 | 1.76 | 3.27 |
| **team 5** | -0.26 | 0.93 | -0.35 | 0.80 | -0.27 | 0.94 | 0.22 | 1.05 | -0.35 | 0.88 | -0.47 | 2.34 |
| **team 6** | 0.08 | 0.80 | 0.15 | 0.92 | 0.07 | 0.72 | -0.13 | 1.03 | -0.10 | 0.55 | -0.07 | 1.84 |
| **team 7** | -0.05 | 0.67 | -0.34 | 0.55 | -0.05 | 0.62 | -0.11 | 0.75 | -0.07 | 0.67 | 0.49 | 1.89 |
| **team 8** | -0.13 | 0.71 | 0.30 | 0.82 | -0.11 | 0.74 | -0.04 | 1.06 | -0.20 | 0.70 | -0.60 | 1.97 |
| **team 9** | 0.36 | 1.76 | 0.41 | 1.35 | 0.09 | 1.66 | 1.12 | 0.87 | 0.13 | 1.66 | -0.49 | 3.43 |
| **team 10** | -0.17 | 0.71 | 0.11 | 0.86 | -0.18 | 0.66 | 0.06 | 0.77 | -0.25 | 0.58 | -0.32 | 1.94 |
| **team 11** | -0.31 | 0.60 | 0.71 | 1.10 | -0.21 | 0.65 | -0.28 | 1.22 | -0.38 | 0.57 | -1.68 | 2.28 |
| **team 12** | -0.47 | 0.54 | 0.56 | 0.88 | -0.54 | 0.55 | 0.70 | 0.92 | -0.40 | 0.45 | -1.37 | 1.72 |
| **team 13** | -0.13 | 0.62 | 0.21 | 0.74 | -0.20 | 0.59 | 0.30 | 0.72 | -0.29 | 0.52 | -0.91 | 1.68 |
| **team 14** | -0.06 | 0.86 | -0.05 | 1.04 | -0.02 | 0.92 | -0.19 | 0.76 | 0.07 | 1.08 | 0.44 | 2.23 |
| **team 15** | -0.89 | 0.74 | 0.59 | 0.71 | -0.87 | 0.62 | 0.40 | 0.89 | -0.85 | 0.55 | -2.81 | 1.97 |
| **team 16** | 0.14 | 0.73 | -0.09 | 0.67 | 0.08 | 0.71 | 0.13 | 0.95 | 0.04 | 0.58 | 0.48 | 1.82 |
| **team 17** | 0.57 | 1.06 | -0.91 | 0.79 | 0.83 | 1.09 | -1.28 | 0.64 | 0.79 | 1.02 | 2.78 | 2.14 |
| **team 18** | -0.54 | 0.66 | 0.76 | 0.68 | -0.60 | 0.55 | 0.52 | 0.66 | -0.73 | 0.45 | -2.41 | 1.70 |
