# Supplementary material for: Is ball-possession style more physically demanding than counter-attacking? The influence of playing style on match performance in professional soccer
Source: Front Psychol. 2023 Jul 7;14:1197039. doi: 10.3389/fpsyg.2023.1197039 (PMC10361297; doi:10.3389/fpsyg.2023.1197039)
Supplement: Supplementary file 3 [file Table_3.DOCX]

# **Supplementary Table 3.** Means ± SD for the parameters (total values) included in the formula for the playing style coefficient [PSC] for every team.

|  | **Passes per action** | | **Forward passing** | | **Target player passes** | | **Passing success rate** | | **Forward passing success rate** | | **Ball possession rate** | |
| --- | --- | --- | --- | --- | --- | --- | --- | --- | --- | --- | --- | --- |
|  | **mean** | **SD** | **mean** | **SD** | **mean** | **SD** | **mean** | **SD** | **mean** | **SD** | **mean** | **SD** |
| **team 1** | 6.63 | 1.76 | 0.58 | 0.03 | 0.98 | 0.01 | 0.86 | 0.05 | 0.82 | 0.06 | 0.59 | 0.15 |
| **team 2** | 5.64 | 1.56 | 0.58 | 0.04 | 0.98 | 0.01 | 0.84 | 0.05 | 0.79 | 0.07 | 0.53 | 0.17 |
| **team 3** | 5.49 | 0.68 | 0.57 | 0.03 | 0.98 | 0.01 | 0.86 | 0.03 | 0.81 | 0.04 | 0.60 | 0.17 |
| **team 4** | 5.99 | 2.50 | 0.58 | 0.03 | 0.98 | 0.00 | 0.85 | 0.03 | 0.80 | 0.05 | 0.65 | 0.15 |
| **team 5** | 4.44 | 1.32 | 0.58 | 0.03 | 0.97 | 0.01 | 0.80 | 0.04 | 0.74 | 0.05 | 0.54 | 0.15 |
| **team 6** | 4.82 | 0.82 | 0.57 | 0.03 | 0.98 | 0.01 | 0.81 | 0.05 | 0.76 | 0.06 | 0.45 | 0.11 |
| **team 7** | 4.86 | 1.01 | 0.60 | 0.02 | 0.98 | 0.01 | 0.83 | 0.05 | 0.79 | 0.07 | 0.55 | 0.12 |
| **team 8** | 4.66 | 1.05 | 0.56 | 0.03 | 0.97 | 0.01 | 0.80 | 0.05 | 0.73 | 0.07 | 0.46 | 0.16 |
| **team 9** | 5.16 | 2.50 | 0.58 | 0.02 | 0.97 | 0.01 | 0.80 | 0.04 | 0.73 | 0.05 | 0.44 | 0.17 |
| **team 10** | 4.58 | 0.88 | 0.58 | 0.03 | 0.98 | 0.01 | 0.82 | 0.04 | 0.75 | 0.07 | 0.51 | 0.16 |
| **team 11** | 4.38 | 0.86 | 0.57 | 0.03 | 0.97 | 0.01 | 0.75 | 0.07 | 0.68 | 0.08 | 0.35 | 0.16 |
| **team 12** | 4.36 | 0.68 | 0.58 | 0.03 | 0.97 | 0.01 | 0.80 | 0.05 | 0.73 | 0.06 | 0.42 | 0.14 |
| **team 13** | 4.53 | 0.78 | 0.57 | 0.03 | 0.97 | 0.01 | 0.79 | 0.04 | 0.73 | 0.05 | 0.45 | 0.14 |
| **team 14** | 5.07 | 1.62 | 0.60 | 0.03 | 0.98 | 0.01 | 0.83 | 0.03 | 0.78 | 0.04 | 0.53 | 0.16 |
| **team 15** | 3.69 | 0.83 | 0.61 | 0.03 | 0.97 | 0.01 | 0.73 | 0.06 | 0.67 | 0.07 | 0.38 | 0.16 |
| **team 16** | 5.02 | 0.88 | 0.57 | 0.03 | 0.98 | 0.01 | 0.84 | 0.04 | 0.78 | 0.05 | 0.54 | 0.13 |
| **team 17** | 6.16 | 1.54 | 0.58 | 0.02 | 0.98 | 0.01 | 0.87 | 0.03 | 0.83 | 0.04 | 0.68 | 0.11 |
| **team 18** | 3.87 | 0.68 | 0.58 | 0.03 | 0.97 | 0.01 | 0.75 | 0.06 | 0.68 | 0.07 | 0.35 | 0.13 |
|  | **Distance per attack** | | **Relative attacking time** | | **Mean attacking time** | | **Running distance in relation to the time of an attack** | | **Mean passes per attack** | | **PSC** | |
|  | **mean** | **SD** | **mean** | **SD** | **mean** | **SD** | **mean** | **SD** | **mean** | **SD** | **mean** | **SD** |
| **team 1** | 478.12 | 121.08 | -5.89 | 6.36 | 19.64 | 5.81 | 24.71 | 1.63 | 6.63 | 1.76 | 2.76 | 2.82 |
| **team 2** | 399.19 | 109.54 | -1.79 | 6.27 | 16.05 | 5.02 | 25.19 | 1.83 | 5.64 | 1.56 | 1.11 | 2.66 |
| **team 3** | 389.67 | 49.63 | -1.85 | 5.76 | 15.31 | 1.92 | 25.47 | 0.94 | 5.49 | 0.68 | 1.33 | 1.22 |
| **team 4** | 426.99 | 155.30 | -4.51 | 7.90 | 16.73 | 7.01 | 25.96 | 1.41 | 5.99 | 2.50 | 1.76 | 3.27 |
| **team 5** | 348.93 | 95.19 | -2.38 | 5.53 | 13.39 | 4.33 | 26.36 | 1.88 | 4.44 | 1.32 | -0.47 | 2.34 |
| **team 6** | 383.94 | 82.39 | 1.01 | 6.30 | 14.97 | 3.33 | 25.75 | 1.85 | 4.82 | 0.82 | -0.07 | 1.84 |
| **team 7** | 370.36 | 69.04 | -2.35 | 3.77 | 14.42 | 2.86 | 25.77 | 1.34 | 4.86 | 1.01 | 0.49 | 1.89 |
| **team 8** | 362.49 | 72.88 | 2.04 | 5.67 | 14.15 | 3.40 | 25.90 | 1.90 | 4.66 | 1.05 | -0.60 | 1.97 |
| **team 9** | 412.15 | 180.81 | 2.80 | 9.26 | 15.06 | 7.66 | 27.98 | 1.55 | 5.16 | 2.50 | -0.49 | 3.43 |
| **team 10** | 358.46 | 72.89 | 0.75 | 5.91 | 13.82 | 3.05 | 26.07 | 1.38 | 4.58 | 0.88 | -0.32 | 1.94 |
| **team 11** | 344.02 | 61.57 | 4.88 | 7.58 | 13.68 | 2.99 | 25.46 | 2.18 | 4.38 | 0.86 | -1.68 | 2.28 |
| **team 12** | 327.82 | 55.07 | 3.87 | 6.03 | 12.14 | 2.53 | 27.22 | 1.65 | 4.36 | 0.68 | -1.37 | 1.72 |
| **team 13** | 361.85 | 63.93 | 1.42 | 5.10 | 13.72 | 2.74 | 26.51 | 1.29 | 4.53 | 0.78 | -0.91 | 1.68 |
| **team 14** | 369.62 | 88.37 | -0.37 | 7.14 | 14.55 | 4.25 | 25.64 | 1.36 | 5.07 | 1.62 | 0.44 | 2.23 |
| **team 15** | 283.99 | 75.72 | 4.07 | 4.88 | 10.66 | 2.86 | 26.69 | 1.59 | 3.69 | 0.83 | -2.81 | 1.97 |
| **team 16** | 390.28 | 74.47 | -0.63 | 4.63 | 15.01 | 3.26 | 26.20 | 1.70 | 5.02 | 0.88 | 0.48 | 1.82 |
| **team 17** | 433.83 | 108.59 | -6.29 | 5.42 | 18.46 | 5.05 | 23.68 | 1.15 | 6.16 | 1.54 | 2.78 | 2.14 |
| **team 18** | 319.84 | 67.15 | 5.23 | 4.70 | 11.90 | 2.55 | 26.91 | 1.18 | 3.87 | 0.68 | -2.41 | 1.70 |
