# Supplementary material for: Is ball-possession style more physically demanding than counter-attacking? The influence of playing style on match performance in professional soccer
Source: Front Psychol. 2023 Jul 7;14:1197039. doi: 10.3389/fpsyg.2023.1197039 (PMC10361297; doi:10.3389/fpsyg.2023.1197039)
Supplement: Supplementary file 4 [file Table_4.DOCX]

# **Supplementary Table 4.** Means ± SD for the technical, the physical, and the success parameters for every team.

|  | **accelerations** | | **accelerations in relation to time** | | **decelerations** | | **decelerations in relation to time** | | **sprinting distance** | | **sprinting distance in relation to time** | |
| --- | --- | --- | --- | --- | --- | --- | --- | --- | --- | --- | --- | --- |
|  | **mean** | **SD** | **mean** | **SD** | **mean** | **SD** | **mean** | **SD** | **mean** | **SD** | **mean** | **SD** |
| **team 1** | 374.59 | 150.18 | 18.91 | 3.56 | 397.18 | 170.12 | 20.04 | 4.49 | 450.00 | 173.87 | 24.79 | 10.73 |
| **team 2** | 263.65 | 92.43 | 16.63 | 3.50 | 285.41 | 105.44 | 17.94 | 3.83 | 286.67 | 155.36 | 19.17 | 10.58 |
| **team 3** | 324.76 | 85.33 | 21.40 | 5.82 | 344.59 | 89.64 | 22.71 | 6.26 | 395.99 | 210.61 | 26.40 | 15.35 |
| **team 4** | 408.71 | 280.89 | 23.37 | 7.08 | 454.35 | 327.26 | 25.82 | 8.53 | 329.94 | 136.85 | 22.01 | 12.45 |
| **team 5** | 229.76 | 101.38 | 17.05 | 4.07 | 272.24 | 131.36 | 20.01 | 4.85 | 344.98 | 193.91 | 26.42 | 15.02 |
| **team 6** | 205.06 | 52.90 | 13.80 | 2.52 | 231.06 | 49.98 | 15.62 | 2.37 | 327.59 | 159.66 | 22.61 | 11.28 |
| **team 7** | 256.47 | 79.04 | 17.65 | 3.70 | 285.71 | 94.31 | 19.60 | 4.49 | 371.07 | 130.00 | 26.70 | 11.14 |
| **team 8** | 238.76 | 90.15 | 16.96 | 5.02 | 258.82 | 105.54 | 18.25 | 5.78 | 317.60 | 152.04 | 23.90 | 14.03 |
| **team 9** | 232.41 | 139.65 | 15.30 | 3.49 | 280.24 | 210.39 | 17.54 | 4.44 | 283.70 | 116.23 | 22.18 | 11.08 |
| **team 10** | 215.47 | 59.05 | 15.74 | 3.99 | 247.12 | 63.01 | 18.02 | 3.47 | 287.69 | 147.63 | 21.48 | 12.37 |
| **team 11** | 178.41 | 66.18 | 13.15 | 4.12 | 188.35 | 77.79 | 13.77 | 4.78 | 186.55 | 68.50 | 14.35 | 6.24 |
| **team 12** | 189.88 | 54.53 | 16.16 | 4.98 | 205.41 | 59.30 | 17.44 | 5.35 | 285.00 | 130.20 | 24.46 | 12.48 |
| **team 13** | 226.12 | 63.25 | 16.52 | 3.58 | 245.18 | 82.25 | 17.82 | 4.83 | 299.12 | 116.70 | 22.87 | 11.24 |
| **team 14** | 271.00 | 129.92 | 18.25 | 4.47 | 289.06 | 122.93 | 19.63 | 4.60 | 344.43 | 121.21 | 24.42 | 9.04 |
| **team 15** | 149.88 | 38.13 | 14.36 | 3.14 | 172.76 | 47.30 | 16.43 | 3.34 | 242.70 | 118.30 | 22.99 | 9.52 |
| **team 16** | 286.82 | 78.24 | 19.27 | 4.29 | 303.12 | 85.21 | 20.40 | 5.09 | 281.24 | 96.91 | 19.51 | 7.19 |
| **team 17** | 379.00 | 102.79 | 21.01 | 5.11 | 403.65 | 102.90 | 22.31 | 4.58 | 367.64 | 137.11 | 21.71 | 12.10 |
| **team 18** | 168.06 | 63.89 | 13.93 | 4.07 | 194.94 | 78.71 | 16.13 | 5.02 | 241.17 | 88.84 | 20.81 | 7.77 |
|  | **high-intensity distance** | | **high-intensity distance in relation to time** | | **percentage short passes** | | **success rate passes short** | | **percentage medium passes** | | **success rate passes medium** | |
|  | **mean** | **SD** | **mean** | **SD** | **mean** | **SD** | **mean** | **SD** | **mean** | **SD** | **mean** | **SD** |
| **team 1** | 1116.13 | 280.40 | 59.48 | 14.01 | 0.38 | 0.06 | 1.11 | 0.05 | 0.55 | 0.06 | 1.15 | 0.06 |
| **team 2** | 810.49 | 323.94 | 54.08 | 23.81 | 0.41 | 0.06 | 1.10 | 0.04 | 0.52 | 0.06 | 1.21 | 0.08 |
| **team 3** | 962.72 | 307.41 | 63.28 | 20.09 | 0.37 | 0.08 | 1.08 | 0.02 | 0.55 | 0.06 | 1.15 | 0.04 |
| **team 4** | 1034.04 | 374.63 | 65.41 | 22.13 | 0.39 | 0.09 | 1.10 | 0.04 | 0.55 | 0.10 | 1.18 | 0.06 |
| **team 5** | 820.57 | 284.83 | 62.94 | 19.86 | 0.30 | 0.09 | 1.17 | 0.07 | 0.58 | 0.11 | 1.23 | 0.07 |
| **team 6** | 741.43 | 256.52 | 50.39 | 16.42 | 0.33 | 0.08 | 1.14 | 0.04 | 0.55 | 0.08 | 1.18 | 0.08 |
| **team 7** | 878.42 | 206.42 | 61.06 | 9.04 | 0.46 | 0.10 | 1.12 | 0.05 | 0.46 | 0.09 | 1.21 | 0.09 |
| **team 8** | 750.43 | 272.24 | 55.39 | 22.07 | 0.33 | 0.09 | 1.13 | 0.05 | 0.55 | 0.10 | 1.21 | 0.08 |
| **team 9** | 874.64 | 289.62 | 63.59 | 19.40 | 0.39 | 0.12 | 1.13 | 0.06 | 0.50 | 0.12 | 1.24 | 0.10 |
| **team 10** | 800.28 | 222.33 | 59.20 | 16.88 | 0.35 | 0.09 | 1.12 | 0.05 | 0.54 | 0.07 | 1.20 | 0.07 |
| **team 11** | 530.71 | 129.86 | 40.17 | 11.46 | 0.27 | 0.06 | 1.21 | 0.08 | 0.57 | 0.08 | 1.29 | 0.18 |
| **team 12** | 819.26 | 254.89 | 69.43 | 20.19 | 0.43 | 0.07 | 1.12 | 0.04 | 0.44 | 0.05 | 1.27 | 0.10 |
| **team 13** | 844.18 | 248.70 | 62.58 | 18.66 | 0.37 | 0.09 | 1.16 | 0.08 | 0.53 | 0.10 | 1.23 | 0.09 |
| **team 14** | 902.24 | 263.93 | 63.13 | 16.18 | 0.40 | 0.08 | 1.13 | 0.04 | 0.52 | 0.08 | 1.19 | 0.06 |
| **team 15** | 611.73 | 263.96 | 56.89 | 19.88 | 0.33 | 0.08 | 1.20 | 0.08 | 0.54 | 0.10 | 1.35 | 0.10 |
| **team 16** | 940.77 | 232.25 | 64.55 | 18.23 | 0.45 | 0.11 | 1.10 | 0.04 | 0.46 | 0.11 | 1.21 | 0.10 |
| **team 17** | 994.76 | 201.16 | 56.93 | 17.76 | 0.36 | 0.07 | 1.10 | 0.02 | 0.58 | 0.07 | 1.14 | 0.05 |
| **team 18** | 607.42 | 207.80 | 51.41 | 16.00 | 0.38 | 0.10 | 1.15 | 0.05 | 0.45 | 0.11 | 1.30 | 0.11 |
|  | **percentage long passes** | | **success rate passes long** | | **percentage horizontal passes** | | **success rate passes horizontally** | | **percentage backward passes** | | **success rate passes backward** | |
|  | **mean** | **SD** | **mean** | **SD** | **mean** | **SD** | **mean** | **SD** | **mean** | **SD** | **mean** | **SD** |
| **team 1** | 0.07 | 0.03 | 1.87 | 0.41 | 0.52 | 0.05 | 0.95 | 0.03 | 0.13 | 0.01 | 0.90 | 0.04 |
| **team 2** | 0.07 | 0.04 | 2.34 | 0.78 | 0.49 | 0.06 | 0.93 | 0.04 | 0.13 | 0.02 | 0.90 | 0.03 |
| **team 3** | 0.08 | 0.03 | 2.13 | 0.51 | 0.50 | 0.05 | 0.96 | 0.02 | 0.15 | 0.02 | 0.91 | 0.03 |
| **team 4** | 0.06 | 0.02 | 2.44 | 0.66 | 0.50 | 0.05 | 0.95 | 0.03 | 0.14 | 0.02 | 0.89 | 0.03 |
| **team 5** | 0.12 | 0.03 | 1.94 | 0.42 | 0.48 | 0.06 | 0.93 | 0.04 | 0.13 | 0.02 | 0.87 | 0.04 |
| **team 6** | 0.11 | 0.04 | 2.46 | 0.54 | 0.44 | 0.05 | 0.95 | 0.03 | 0.14 | 0.02 | 0.89 | 0.03 |
| **team 7** | 0.08 | 0.04 | 2.43 | 0.68 | 0.47 | 0.06 | 0.96 | 0.03 | 0.13 | 0.02 | 0.89 | 0.04 |
| **team 8** | 0.12 | 0.04 | 2.45 | 0.68 | 0.44 | 0.06 | 0.91 | 0.04 | 0.13 | 0.02 | 0.89 | 0.04 |
| **team 9** | 0.11 | 0.04 | 2.53 | 0.54 | 0.44 | 0.05 | 0.95 | 0.03 | 0.14 | 0.02 | 0.88 | 0.03 |
| **team 10** | 0.11 | 0.05 | 2.41 | 0.63 | 0.43 | 0.06 | 0.93 | 0.04 | 0.14 | 0.02 | 0.89 | 0.03 |
| **team 11** | 0.16 | 0.05 | 2.41 | 0.58 | 0.39 | 0.07 | 0.93 | 0.04 | 0.14 | 0.02 | 0.84 | 0.08 |
| **team 12** | 0.13 | 0.04 | 2.17 | 0.39 | 0.44 | 0.06 | 0.94 | 0.03 | 0.13 | 0.02 | 0.87 | 0.03 |
| **team 13** | 0.10 | 0.04 | 2.99 | 0.67 | 0.44 | 0.05 | 0.93 | 0.05 | 0.12 | 0.02 | 0.88 | 0.02 |
| **team 14** | 0.08 | 0.03 | 2.40 | 0.47 | 0.47 | 0.04 | 0.93 | 0.03 | 0.13 | 0.02 | 0.89 | 0.03 |
| **team 15** | 0.13 | 0.05 | 3.08 | 1.29 | 0.38 | 0.06 | 0.94 | 0.06 | 0.11 | 0.02 | 0.83 | 0.06 |
| **team 16** | 0.10 | 0.03 | 2.01 | 0.44 | 0.46 | 0.05 | 0.96 | 0.02 | 0.15 | 0.02 | 0.89 | 0.04 |
| **team 17** | 0.07 | 0.02 | 1.83 | 0.50 | 0.52 | 0.04 | 0.95 | 0.03 | 0.14 | 0.01 | 0.91 | 0.02 |
| **team 18** | 0.17 | 0.05 | 2.85 | 0.92 | 0.41 | 0.05 | 0.92 | 0.05 | 0.12 | 0.02 | 0.86 | 0.05 |
|  | **passing velocity** | | **dribblings** | | **success rate dribblings** | | **x goals** | | **goals** | | **points** | |
|  | **mean** | **SD** | **mean** | **SD** | **mean** | **SD** | **mean** | **SD** | **mean** | **SD** | **mean** | **SD** |
| **team 1** | 50.88 | 1.61 | 9.94 | 2.75 | 0.72 | 0.14 | 1.81 | 0.78 | 2.24 | 0.75 | 2.24 | 1.25 |
| **team 2** | 48.16 | 1.49 | 9.00 | 4.57 | 0.69 | 0.22 | 1.36 | 0.97 | 1.71 | 1.45 | 1.18 | 1.42 |
| **team 3** | 51.51 | 1.51 | 16.71 | 6.27 | 0.62 | 0.13 | 1.18 | 0.70 | 1.18 | 1.42 | 1.18 | 1.29 |
| **team 4** | 48.59 | 1.14 | 6.94 | 4.51 | 0.66 | 0.28 | 1.70 | 0.70 | 1.65 | 1.17 | 1.71 | 1.45 |
| **team 5** | 52.61 | 2.37 | 7.71 | 4.04 | 0.55 | 0.17 | 1.20 | 0.31 | 1.82 | 1.13 | 1.88 | 1.41 |
| **team 6** | 49.39 | 1.99 | 8.18 | 3.78 | 0.63 | 0.20 | 1.04 | 0.61 | 1.12 | 1.27 | 1.35 | 1.46 |
| **team 7** | 49.45 | 1.24 | 8.94 | 5.03 | 0.63 | 0.21 | 1.54 | 0.85 | 2.00 | 1.22 | 1.82 | 1.33 |
| **team 8** | 52.83 | 1.89 | 11.76 | 3.77 | 0.64 | 0.16 | 1.12 | 0.64 | 0.94 | 1.03 | 1.00 | 1.22 |
| **team 9** | 50.19 | 2.28 | 7.00 | 4.50 | 0.58 | 0.29 | 1.27 | 0.78 | 0.94 | 0.90 | 1.47 | 1.23 |
| **team 10** | 51.21 | 1.91 | 10.53 | 5.50 | 0.68 | 0.18 | 1.24 | 0.65 | 0.94 | 1.03 | 1.00 | 1.37 |
| **team 11** | 52.00 | 1.97 | 7.88 | 3.79 | 0.63 | 0.20 | 0.98 | 0.70 | 0.59 | 0.87 | 1.00 | 1.22 |
| **team 12** | 49.02 | 1.82 | 7.59 | 2.85 | 0.58 | 0.23 | 0.95 | 0.43 | 0.88 | 0.93 | 0.88 | 1.11 |
| **team 13** | 50.32 | 1.61 | 10.71 | 6.27 | 0.68 | 0.20 | 0.74 | 0.70 | 0.65 | 1.06 | 0.53 | 1.01 |
| **team 14** | 48.42 | 1.46 | 11.06 | 7.24 | 0.74 | 0.20 | 1.12 | 0.76 | 1.29 | 1.21 | 1.24 | 1.39 |
| **team 15** | 49.28 | 1.84 | 7.41 | 3.02 | 0.59 | 0.19 | 1.27 | 0.68 | 1.24 | 1.15 | 1.71 | 1.31 |
| **team 16** | 50.61 | 1.83 | 9.41 | 5.48 | 0.62 | 0.21 | 1.39 | 0.71 | 1.35 | 1.17 | 1.65 | 1.37 |
| **team 17** | 50.08 | 1.55 | 13.41 | 7.42 | 0.76 | 0.15 | 2.13 | 0.77 | 2.71 | 1.49 | 2.24 | 1.25 |
| **team 18** | 50.68 | 1.86 | 7.71 | 4.51 | 0.56 | 0.23 | 0.99 | 0.51 | 1.00 | 0.94 | 1.06 | 1.34 |
