# Supplementary figures and images for: Is ball-possession style more physically demanding than counter-attacking? The influence of playing style on match performance in professional soccer
Source: Front Psychol. 2023 Jul 7;14:1197039. doi: 10.3389/fpsyg.2023.1197039 (PMC10361297; doi:10.3389/fpsyg.2023.1197039)

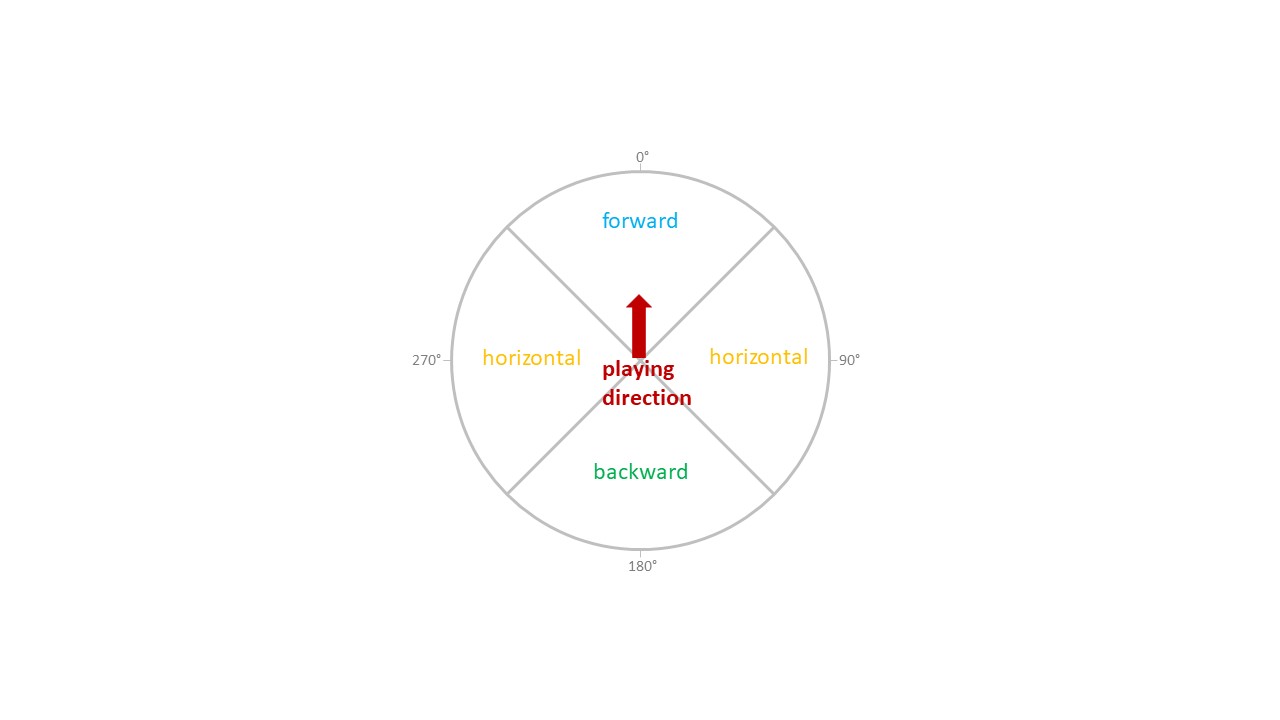

Supplement: Supplementary file 5 [file Image_1.JPEG]
